# Association Between Calcium Phosphate Product and Carotid Intimal Medial Thickness in Patients With Chronic Kidney Disease: A Cross-Sectional Study

**DOI:** 10.7759/cureus.89014

**Published:** 2025-07-29

**Authors:** Priscilla Allam, Faraz Farishta

**Affiliations:** 1 Internal Medicine, Care Hospitals, Hyderabad, IND; 2 Endocrinology, Diabetes and Metabolism, FS Endocrine and Diabetes Center, Hyderabad, IND

**Keywords:** association, calcium phosphate product, cardiovascular disease, carotid intimal medial thickness, chronic kidney disease

## Abstract

Background

Chronic kidney disease has been found to increase the risk of cardiovascular disease (CVD). Carotid intima media thickness (CIMT) is a commonly used surrogate marker for assessing atherosclerosis. Recent interest has emerged in exploring the potential of calcium phosphate product (Ca x Pi) as an alternative or adjunct in evaluating cardiovascular risk in these patients. This study aimed to find the correlation between Ca x Pi and CIMT in patients with advanced chronic kidney disease.

Methods

A cross-sectional study was conducted at Thumbay Hospital New Life, Hyderabad, from January 2020 to December 2020. Fifty patients with advanced chronic kidney disease (stages 3 and above) were included after applying the inclusion and exclusion criteria. Serum Ca x Pi levels were measured along with CIMT using carotid ultrasound. Statistical analysis was performed to assess the correlation between Ca x Pi and CIMT.

Results

The mean Ca x Pi was 44.8±11, and the mean CIMT was 1.68±0.47 mm. A positive correlation was found between Ca x Pi and CIMT (p = 0.03), while serum phosphorus showed a strong correlation with CIMT (p < 0.01).

Conclusion

Ca x Pi shows promise as a surrogate marker for assessing cardiovascular risk in patients with chronic kidney disease, alongside CIMT. Further research, including larger longitudinal studies, is warranted to validate these findings and explore the utility of Ca x Pi in clinical practice.

## Introduction

Chronic kidney disease (CKD) is a significant contributor to global mortality, with 1.2 million deaths reported in 2017 and a rising trend observed since 1990 [1]. CKD is associated with a substantial risk for morbidity, all-cause mortality, and cardiovascular mortality. The association between CKD and cardiovascular disease (CVD) is well-established, with patients at advanced CKD stages facing notably higher risks of CVD-related events [2]. Risk factors such as hypertension, diabetes, and inflammation exacerbate the cardiovascular burden in CKD patients [3].

Carotid artery imaging, particularly carotid intimal medial thickness (CIMT) measured via ultrasonography, serves as a valuable tool for assessing cardiovascular involvement in CKD patients [4]. CIMT not only serves as a surrogate marker for atherosclerosis but also predicts future cardiovascular events [5-6].

While CIMT assessment offers advantages such as safety, non-invasiveness, and cost-effectiveness, exploring additional biomarkers for CVD risk assessment in CKD patients is warranted. Recent attention has focused on calcium-phosphate product (Ca × Pi), a clinical indicator of mineral crystallization associated with vascular calcification [7]. Despite its potential utility in assessing CV risk, Ca × Pi's comparative effectiveness with CIMT remains understudied, particularly in the CKD population. 

This cross-sectional study aims to evaluate the correlation between the calcium-phosphate product (Ca × Pi) and CIMT in patients with advanced chronic kidney disease (CKD) stages 3 and above, potentially providing insights into novel biomarkers for CVD risk assessment.

## Materials and methods

A cross-sectional study design was used to investigate the correlation between serum calcium-phosphate product (Ca × Pi) and CIMT in patients with advanced CKD stages 3 and above. The study was conducted at Thumbay Hospital New Life, Hyderabad, from January 2020 to December 2020. Admitted and outpatient CKD patients were recruited from the Department of General Medicine and Nephrology. This study has been reported in line with the Strengthening the Reporting of Observational Studies in Epidemiology (STROBE) checklist for cross-sectional studies [8].

Inclusion and exclusion criteria

Inclusion criteria comprised adults aged 18-59 years diagnosed with advanced CKD (stages 3 and above). Exclusion criteria included age over 60 years, acute kidney injury, known major cardiovascular events such as myocardial infarction or stroke, history of coronary revascularization, history of primary or secondary bone malignancies, history of thyroid or parathyroid disorders, patients on glucocorticoids, statin therapy, and history of carotid surgery.

Ethical considerations

Following application of inclusion and exclusion criteria over five months, fifty eligible patients were included in the study. Ethical clearance was obtained from the Institutional Ethical Committee. Written informed consent was obtained from all participants before the study commencement. A detailed medical and surgical history was collected from all participants before blood sampling and carotid ultrasound. The history was recorded on a pre-specified data collection form along with other study-related variables.

Variables

The variables collected for the study were: age, sex, body weight, body height, cardiovascular risk factors, history of cardiovascular events, serum calcium, serum phosphorus, blood urea, creatinine, plasma albumin, fasting lipid profile, fasting blood glucose, estimated glomerular filtration rate, and CIMT. Of the several formulas available for the correction of calcium for the serum albumin level, albumin-corrected calcium was estimated with the equation suggested by the Kidney Disease Outcomes Quality Initiative (KDOQI) guidelines [9-11]. First, the corrected serum calcium was calculated using the KDOQI formula to adjust for serum albumin: Corrected Calcium (mg/dl) = Total Calcium (mg/dl) + 0.0704 × [34 − serum albumin (g/L)]. Next, the corrected value of calcium obtained from the above formula was then used in calculating the calcium-phosphate product (Ca × Pi) with the following formula: Corrected Ca-P product = (4 × corrected serum calcium) × (3.1 × serum phosphorus) [12-13]. 

Method

All participants underwent the same protocol of ultrasound imaging. A single sonographer performed all the procedures. The sonographer was blinded to the blood results of the patients. The images were obtained using a Philips HD 11 XE Colour Doppler unit B-mode ultrasound (Koninklijke Philips Electronics N.V., Pune, India) in a carotid Doppler setting, performed with a 10 MHz linear transducer. The patients were positioned supine, with the neck extended and the head turned 45 degrees away from the side of the examination. Longitudinal views were used to image the common carotid arteries. The intima-media thickness of both common carotid arteries was measured at three different sites in the anterior wall between two hyperechoic walls about 1 cm proximal to the site of bifurcation of the common carotid artery. The average of the three measurements was noted as the CIMT measurement of that particular side of the artery. The measurement of the side that was higher was taken as the CIMT for the study. A CIMT measurement of > 0.8 mm was considered thickened for the study [9].

Statistical analysis

Statistical analysis was carried out utilizing R software (The R Foundation for Statistical Computing, Vienna, Austria). Study variables are presented using descriptive statistics; normally distributed continuous variables as mean and standard deviation (SD), and skewed distributions by the median and interquartile range (IQR). Binary and categorical variables are presented using counts and percentages. Correlation between the calcium phosphorus product and CIMT was assessed by the Pearson correlation test. P values < 0.05 were considered statistically significant.

## Results

A total of 50 patients were studied, among whom the majority were males, comprising 26 (52%) patients. The average age of the patients was 49.8±5.07 years. The demographic and anthropometric distribution is depicted in the table below (Table 1).

**Table 1:** Demographic Profile of Patients (n = 50)

GENDER (n. %)	*FREQUENCY (PERCENTAGE)/Mean *± *SD*
Male	26 (52%)
Female	24 (48%)
EDUCATION (n. %)	
Illiterate	8 (16%)
School Education	26 (52%)
Intermediate	2 (4%)
College Education	2 (4%)
ANTHROPOMETRY {Mean ± SD}	
Height (cm)	161.3 ± 8.7 (cm)
Weight (kgs)	57.5 ± 18.7 (kgs)
Body Mass Index (kg/m^2^)	21.7 ± 5.7 (kg/m^2^)

**Figure 1:**
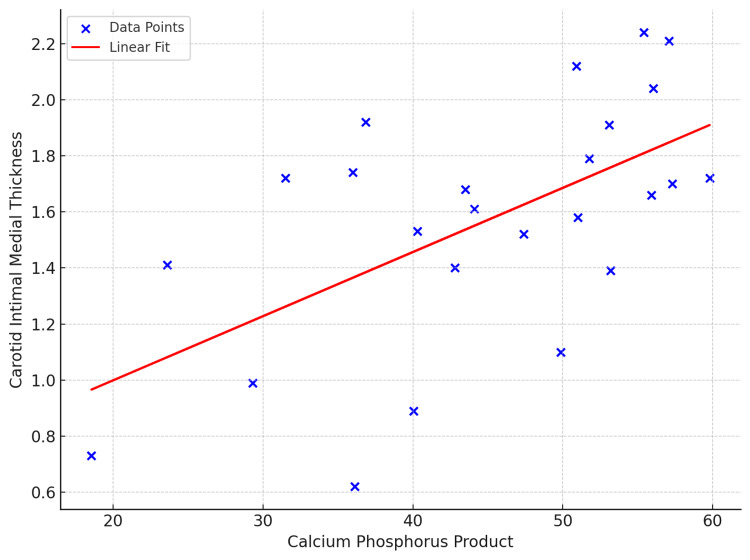
Correlation Between CIMT and Calcium-Phosphate Product Scatter plot showing the relationship between the calcium phosphorus product and CIMT. Each blue dot represents an individual patient's data point. A red line indicates the linear regression trend, suggesting a positive correlation between the two variables. CIMT is measured in millimeters. CIMT: carotid intimal medial thickness.

The scatter plot (Figure 1) below demonstrates the relationship between the calcium phosphorus product and CIMT among study participants. Each blue point represents an individual patient's data, with the X-axis denoting the Calcium Phosphorus Product values and the Y-axis indicating corresponding CIMT measurements.

A linear regression line (red) is drawn across to highlight the trend in the data. The positive slope of this line suggests a moderate positive correlation: the calcium phosphorus product increases, and there is a tendency for the CIMT values to rise as well. This implies that vascular calcification, indicated by elevated calcium-phosphorus levels, is associated with increased arterial wall thickness, a surrogate marker for subclinical atherosclerosis.

**Figure 2:**
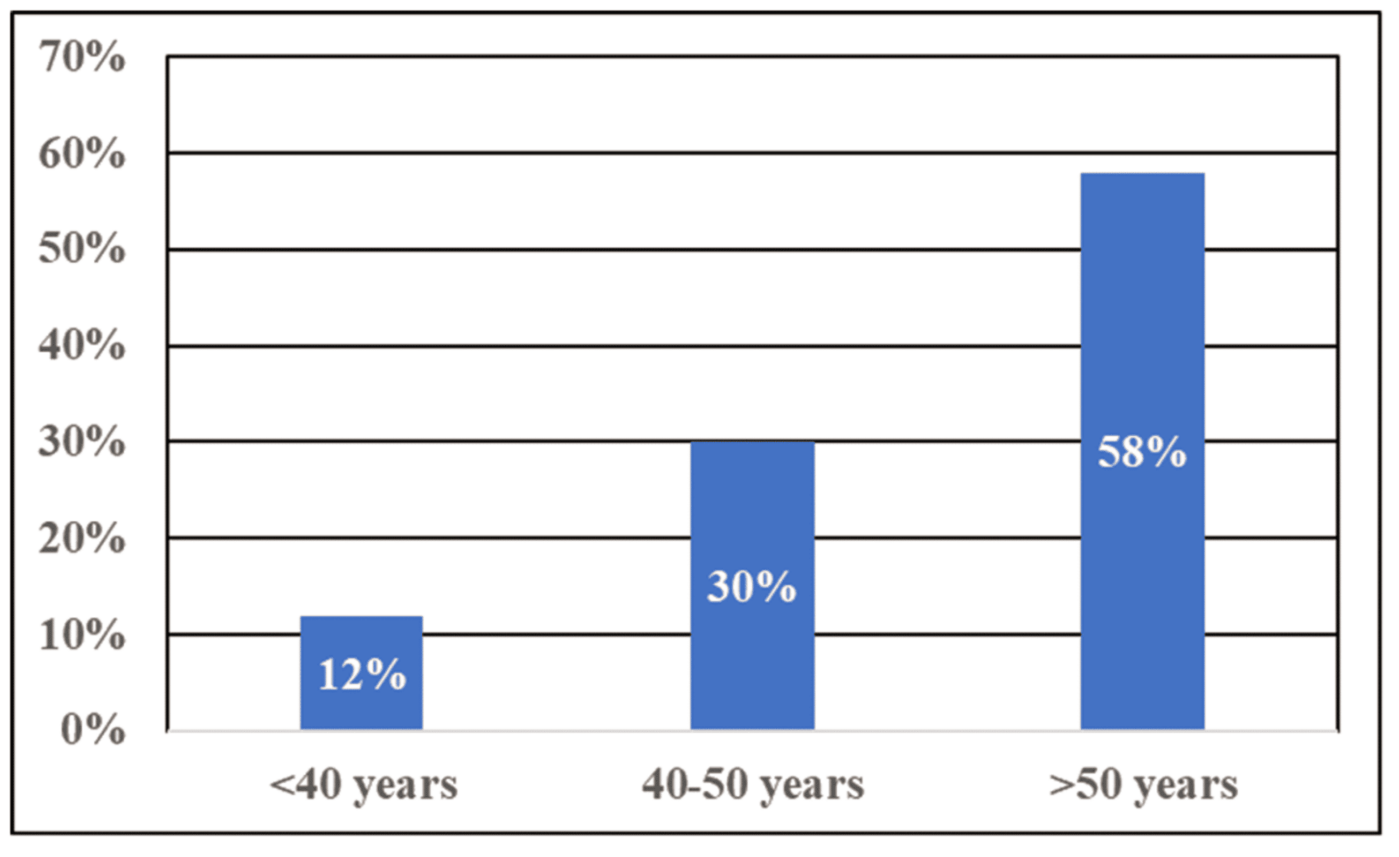
Age-Wise Categorization of the Patients

The age groups were classified into different categories and shown in a bar graph (Figure 2).

The duration of renal dysfunction ranged from three years to 10 years. Two (4%) patients reported symptoms suggestive of angina but had not undergone a cardiac evaluation. More than half of the patients had a family history of CVD, which was present in 14 (28%). The majority of the patients were hypertensive, seen in 42 (84%), and diabetic, seen in 18 (36%). The cardiovascular risk factors are given in Table 2.

**Table 2:** Cardiovascular Risk Factors

CLINICAL VARIABLES	FREQUENCY (PERCENTAGE)
Diabetes	18 (36%)
Hypertension	42 (84%)
Smoking	2 (4%)
Alcohol	8(16%)
Family History of Cardiovascular Disease	14 (28%)
Hyperlipidemia	2 (4%)

The investigations performed are given in Table 3.

**Table 3:** Laboratory Parameters eGFR: estimated glomerular filtration rate.

STUDY VARIABLES (Units)	Mean ± SD
Hemoglobin (gm/dl)	9.12 ± 1.5
Blood Urea (mg/dl)	90.48 ± 38.5
Serum Creatinine (mg/dl)	5.24 ± 2.2
Fasting Blood Glucose (mg/dl)	122.92 ± 32.4
Serum Calcium (mg/dl)	8.24 ± 0.72
Serum Phosphorus (mg/dl)	4.8 ± 1.1
Plasma Albumin (mg/dl)	2.8 ± 0.5
Corrected Calcium (mg/dl)	9.32 ± 0.74
Calcium Phosphate Product	44.88 ± 11
eGFR (ml/min/1.73m2)	15.88 ± 12.5
Tota Cholesterol (mg/dl)	158.16 ± 39
Serum Triglycerides (mg/dl)	112.64 ± 62.9
Carotid Intima Media Thickness (mm)	1.68 ± 0.47

Correlation between biochemical markers and CIMT

The mean Ca x Pi was 44.8±11, and the mean CIMT was 1.68±0.47 mm. There was a linear correlation between CIMT and Ca x Pi (Figure 1), with p p-value significant for a confidence interval of 95%. The correlation between CIMT and calcium phosphate product variable yielded a positive association with a Pearson correlation coefficient of 0.43 (r = 0.43) and a statistically significant p-value of 0.03. Serum phosphorus showed a strong correlation with CIMT (p < 0.01). However, serum calcium did not correlate with CIMT. Ninety percent of the patients had a CIMT value above 0.8mm.

The statistical significance is shown in Table 4.

**Table 4:** Correlation between Carotid Intima Media Thickness (CIMT) and Calcium Phosphate Product

Variable	Calcium Phosphate Product	p value
Carotid Intima Media Thickness	0.43	0.03

## Discussion

Our findings suggest a significant association between Ca x Pi and CIMT in patients with advanced CKD. While previous studies have explored the relationship between serum phosphate and CIMT, this study adds to the literature by specifically investigating Ca x Pi as a potential biomarker for cardiovascular risk assessment in CKD patients [14-15]. Our study sought to assess the correlation between CIMT as assessed by carotid ultrasound and Ca x Pi in patients with advanced CKD and found a positive correlation between the two. While serum calcium showed no correlation, serum phosphorus was strongly associated with CIMT. Previous studies have assessed the association of serum phosphate with CIMT in patients with CKD and found a strong correlation between the two. Multiple studies have used CIMT as a surrogate marker of CV events, including mortality. In parallel, the role of Ca x Pi as an independent risk predictor for CVD in CKD patients has also been examined.

Studies by Block et al. [16-17] and Ganesh et al. [18] are large registry studies that indicated that hyperphosphatemia and an increased Ca x Pi product were independent risk factors for the survival of dialysis patients. In our study, serum phosphorus levels correlated well with CIMT. Though phosphorus levels were well controlled, their positive association with CIMT suggests that even normal or slightly abnormal values may predict CVD. In addition, the Ca x Pi showed a strong association with CIMT. The mean Ca X Pi was well within the recommended levels by KDOQI. Yet, a positive correlation was seen with Ca x Pi levels with CIMT. The majority of the patients (90%) had high values of CIMT, suggesting that the study population was at increased risk for CVD. Menon et al. [19] studied 840 participants from the randomized cohort of the modification of diet in renal disease study. Their objective was to investigate the relationships of phosphorus and Ca x Pi with outcomes in patients with CKD stages 3 to 5.

Our study is limited by its cross-sectional design, which restricts our ability to establish causality and track changes over time. Additionally, the small sample size and single-center recruitment limit the generalizability of our findings. Furthermore, the extrapolation of our results to other populations may be challenged by variations in social, ethnic, and healthcare system characteristics, as well as differences in nutritional habits. These limitations show the need for larger, multicenter studies to validate our findings across diverse populations and settings. This study lays a foundation for studying the role of Ca x Pi as an alternative or an adjunct to CIMT in risk stratification of CVD in CKD patients. Further, well-controlled longitudinal studies considering the multiple factors associated with mineral metabolism either independently or in combination as a CVD risk assessment tool may shed light on the subject. 

## Conclusions

The correlation between carotid CIMT and CVD risk factors is well-established, indicating its potential as a predictive biomarker. In patients with advanced chronic kidney disease (CKD), our study reveals a significant association between calcium-phosphate product (Ca × Pi) and CIMT, suggesting Ca × Pi's potential as a surrogate marker for assessing CVD risk in this population. However, further investigation through well-controlled prospective studies is needed to confirm these findings and determine whether Ca × Pi alone or in conjunction with other mineral metabolism factors can effectively predict CVD risk in high-risk CKD patients. If validated, Ca × Pi could serve as a valuable tool, particularly in settings where access to carotid ultrasound or skilled sonographers is limited. Additionally, a call for epidemiological or multicenter studies in India is needed to tailor the guidelines to address the specific needs of the local population. Overall, Ca × Pi holds promise as a complementary marker alongside CIMT in assessing cardiovascular risk in CKD patients, emphasizing the necessity for future research to explore its clinical utility further.
